# Anatomy-Based Assessment of Spinal Posture Using IMU Sensors and Machine Learning

**DOI:** 10.3390/s25195963

**Published:** 2025-09-25

**Authors:** Rabia Koca, Yavuz Bahadır Koca

**Affiliations:** 1 Department of Physical Therapy and Rehabilitation, Faculty of Health Sciences, Afyonkarahisar Health Sciences University, 03030 Afyonkarahisar, Türkiye; rinerkoca@gmail.com; 2 Department of Electrical Engineering, Faculty of Engineering, Afyon Kocatepe University, 03200 Afyonkarahisar, Türkiye

**Keywords:** anatomic posture analysis, IMU sensors, machine learning, cervical lordosis, thoracic kyphosis, lumbar lordosis, scoliosis

## Abstract

**Highlights:**

**What are the main findings?**

- IMU-based posture angles were used to derive proxy deviation labels relative to reference ranges from the literature, and machine learning models predicted these labels using demographic and anthropometric variables.
- Daily posture habits, particularly prolonged desk and smartphone usage, significantly influence deviations in cervical lordosis, thoracic kyphosis, and lumbar lordosis.

**What is the implication of the main finding?**

- The IMU-based monitoring of spinal posture may motivate future studies on preventive health strategies, but current findings remain exploratory and underpowered.
- Integrating sensor-based posture monitoring into daily life and occupational settings could be explored in future research, while emphasizing that it is not yet a validated diagnostic tool.

**Abstract:**

Background: This study used inertial measurement unit (IMU)-based posture angle estimates to define proxy risk labels and investigated whether these labels can be predicted from demographic, anthropometric, and lifestyle variables through machine learning analysis. Methods: Thirty healthy individuals aged 18–25 years were included. Demographic and anthropometric data and information on daily living activities were collected. The IMU sensors were placed at vertebral levels C1, C7, T5, T12, and L5. Participants were instructed to stand in an upright posture, followed by a relaxed daily posture. Anatomic postural changes between these positions were analyzed. Cervical lordosis, thoracic kyphosis, lumbar lordosis, and scoliosis risks were predicted using machine learning algorithms, including Random Forest (RF) and Artificial Neural Networks (ANN). Results: Incorrect postures during desk work and phone use were associated with an increased likelihood of posture-related deviations, such as cervical lordosis, thoracic kyphosis, and lumbar lordosis. Conversely, daily physical activity reduced these deviations. Using LOSO and stratified cross-validation with imbalance handling, balanced accuracies ranged between 0.55 and 0.82 across targets, with majority-class baselines between 0.53 and 0.87. For cervical lordosis risk, RF achieved a 0.82 balanced accuracy (95% CI: 0.74–0.97), while other categories showed a moderate but consistent performance. AUPRC values exceeded baseline levels across all models. Conclusions: IMU-based posture angle estimates can be used to identify posture-related risk categories. In this study, ML models have demonstrated predictive relationships with demographic, anthropometric, and lifestyle variables. These findings provide exploratory evidence based on IMU-derived proxy labels in a small cohort of healthy young adults. They represent exploratory indicators of postural deviation rather than clinical outcomes and may motivate future studies on preventive strategies. Importantly, the results remain underpowered relative to the a priori power targets and should be interpreted qualitatively.

## 1. Introduction

Anatomic posture is a concept that defines an individual’s body position against the force of gravity. Posture, directly related to anatomical structures, can be examined statically and dynamically during activities such as standing, sitting, or moving [1]. Static posture provides the stabilization of joints when stationary, while dynamic posture is a state of movement that requires constant adaptation. The vertebral column provides an upright posture as the basic skeletal structure of the body and plays a vital role in maintaining a healthy posture. The relationship between the vertebrae, the spine’s basic structural units, the intervertebral disks’ shock-absorbing function, and the posture-supporting mechanisms of different muscle groups is necessary for maintaining a healthy posture. Muscles that provide postural stability, especially the erector spine muscle and transversus abdominis muscle, increase the correct alignment and load-carrying capacity of the vertebral column [1,2].

Correct posture is a condition in which the musculoskeletal system is aligned correctly and stress on anatomical structures is reduced, increasing biomechanical efficiency. When the vertebral column is viewed from behind in the correct posture, it is seen in a straight line in the vertical plane. If there is a lateral curvature over 10°, it is defined as scoliosis [3]. However, certain anatomical curves are considered normal when viewed from the side. These curves should be at certain angles. The curvature in the cervical region (cervical lordosis) varies between 35° and 45°, the curvature in the thoracic region (thoracic kyphosis) varies between 20° and 40°, and the curvature in the lumbar region (lumbar lordosis) varies between 40° and 60° [4,5,6].

Today’s lifestyle disrupts the spine’s natural curves due to factors such as prolonged sitting, incorrect bed positions, and lifting heavy loads, and can lead to spinal posture disorders [7,8]. In particular, students’ incorrect sitting postures during studying or listening and prolonged phone and computer use can lead to various health problems in later ages [7,9]. Over time, these disorders can affect the spine and muscular system, causing chronic pain, nerve compression, and movement restrictions, reducing the quality of life [8]. Long-term incorrect sitting positions can increase lumbar lordosis and cause back pain or pain in the cervical and thoracic regions [10]. Therefore, the early diagnosis and treatment of spinal posture disorders are essential to prevent the progression of health problems and preserve life. In this direction, spinal posture analysis is essential for determining and demonstrating treatment approaches specific to many posture problems [11].

Spinal posture analysis diagnoses possible deformities and disorders by evaluating the vertebral positions [12]. Physical examinations of the complaint help to detect posture problems through guidance from the patient and are essential in determining the treatment plan. In clinical practice, spinal posture is commonly assessed using radiographic imaging (e.g., Cobb angles), MRI/ultrasonography for soft-tissue evaluation, and laboratory-based tools such as goniometers, photogrammetry, and opto-electronic motion capture systems, each with practical constraints for repeated, real-life monitoring [13,14]. Traditionally, spinal posture is examined in clinics, routine patient examinations, physiotherapy sessions, or laboratory-based evaluations. However, this traditional distortion is insufficient regarding daily posture measurements and timely feedback. Radiographic assessment is the basic method for analyzing spinal posture [15]. Still, it is not often preferred due to the cost and radiation exposure over repeated uses [16]. The detailed examination of muscle and soft tissue structures with techniques such as MRI and ultrasonography provides a clearer understanding of the origin of posture disorders. These methods are particularly valuable in determining the integration of soft tissues and the proper functioning of muscles [17]. Other laboratory-based methods, such as goniometers, photogrammetric systems, and optoelectronic systems, have not been integrated into routine medical practice due to various limitations [10,18,19].

Recent developments in electronics have increased the interest in using IMUs to record anatomic posture analysis in 3D. Software-developed algorithms can process data from IMU sensors to perform an anatomic posture analysis of a person. This detects posture errors, posture disorders, and movement defects in patients and provides feedback to users, allowing early screening and monitoring [20,21].

IMU sensors have become an essential alternative for posture analysis, offering many advantages over traditional optical motion capture systems, such as portability, a low cost, and a radiation-free structure [22,23]. Their small size and ease of use make IMUs ideal for long-term monitoring and data collection in both clinical and non-clinical settings, allowing for the easy tracking of rehabilitation progress and human performance [24,25]. Offering a complementary option compared to traditional motion capture systems, IMUs are a safer alternative to radiation-involved methods such as X-ray or computed tomography (CT), especially for repeated use in cases of disability or paralysis [25,26]. The non-invasive nature of these sensors allows for accurate and concise stance analysis, increasing user comfort and providing long-term usage data [25]. In addition, their usability in today’s conditions and outside the laboratory environment offers various applications, from outdoor analyses in elderly individuals to human–machine movements in factory environments [22,24,27].

However, accurate IMU use requires ongoing calibration and the mitigation of magnetic disturbances and temperature-related drift, together with careful sensor alignment, to ensure measurement stability across environments [23,26,28,29]. IMU sensors offer a practical solution for preventing occupational musculoskeletal disorders in occupational health and safety with the advantages of portability and a low cost [30].

### Research Gap, Contribution, Objectives, and Hypothesis

Despite their value, the above methods are largely static or laboratory-bound and do not readily capture posture during daily activities. There remains a need for portable, clinic-independent monitoring that enables repeated, radiation-free measurements.

We examine a feasibility framework in healthy young adults where IMU-derived posture angles are transformed into proxy deviation labels using literature-based reference ranges, and we explore whether simple, readily collected variables (demographic, anthropometric, and daily behavior) can predict these labels.

The aims of this study are as follows: (i) derive IMU-based proxy deviation labels for cervical lordosis, thoracic kyphosis, and lumbar lordosis; (ii) quantify associations with daily behaviors and anthropometry; (iii) evaluate cross-validated machine learning models that predict these labels while addressing a small sample size and class imbalance.

Easily collected demographic, anthropometric, and daily behavior variables would show measurable associations with IMU-derived proxy deviation labels and enable better-than-chance cross-validated classification in this cohort.

## 2. Materials and Methods

### 2.1. Study Design and Setting

This study was conducted in a university laboratory setting. This was a cross-sectional observational study conducted in a single session. Ethical approval for the study was received from the scientific research ethics committee of Afyonkarahisar Health Sciences University (approval date: 1 September 2023; approval number: 2023/405). A written informed consent form was obtained from all participants. A total of 30 healthy participants, 15 female and 15 male, aged 18–25, were included. The individuals’ demographic characteristics, anthropometric measurements, and daily living activities were collected via a structured questionnaire.

An a priori power analysis was conducted using G*Power 3.1. Based on Cohen’s h ≈ 0.51, α = 0.05, and power = 0.80, a sample size of approximately 30 was estimated as sufficient to detect only large effects. Required accuracies relative to no skill baselines were calculated for each risk category (Table 1). This indicates that the study was adequately powered for large effects but underpowered for smaller ones. However, the cross-validated results obtained in this study (max. ≈ 0.90 for cervical lordosis) did not reach the a priori thresholds listed in Table 1. Therefore, the ML findings should be presented as underpowered exploratory findings and interpreted qualitatively rather than confirmatory.

IMU sensors were used to perform the anatomic posture analysis. Sensors were placed at the cervical 1 (C1), cervical 7 (C7), thoracic 5 (T5), thoracic 12 (T12), and lumbar 5 (L5) levels of the participants’ vertebral column. The participants were first asked to stand upright and then to move to the postures in which they perform the daily activities they are comfortable with. Postural analyses were performed by observing the angular changes between these two situations.

**Figure 1:**
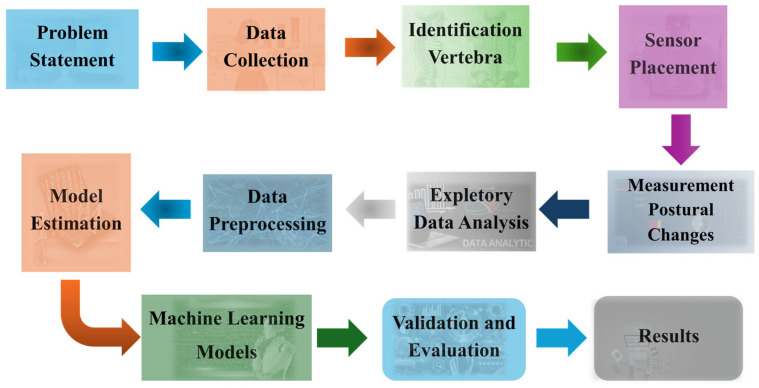
Flow chart summarizing the main stages of the postural analysis and machine learning.

Each participant first stood upright in a standard anatomical position for approximately 10 s. Following this, they were instructed to transition into a relaxed posture that reflected their typical daily posture while standing or sitting. No external correction or constraint was imposed during this transition to ensure natural behavior. Angular data from all five sensors were recorded continuously during both positions, with each recording phase lasting approximately 20 s. The entire procedure took around 5 min per participant, including preparation and calibration. Flexion, extension, right–left lateral flexion, and rotation movements were examined. Data were transferred wirelessly to a central unit via Bluetooth. A heat-map was used only for the visualization of pairwise associations. Deviation/risk labels were not derived from the heat-map; they were defined a priori from IMU-based angles using literature-based reference ranges (cervical lordosis 35–45°, thoracic kyphosis 20–40°, lumbar lordosis 40–60°; scoliosis screening threshold ±10° lateral deviation) [3,4,5,6]. The flow diagram of the study is given in Figure 1.

### 2.2. Participant Criteria and Parameters

Participants were selected according to specified criteria, and their consent was obtained voluntarily, after which their demographic and anthropometric data were collected. Demographic characteristics (age, height, weight, BMI), daily desk sitting time, daily phone/computer use, weekly physical activity, and daily standing time were recorded using a structured questionnaire. The average duration of phone and computer use was primarily collected through self-reported estimates based on participants’ typical weekday routines. To minimize recall bias, participants were encouraged to check screentime statistics or usage-tracking applications on their smartphones. To reduce recall bias, participants were instructed to consider their use over the past seven days and to report average daily usage. Where possible, participants were also encouraged to check screentime statistics or usage-tracking applications on their smartphones to provide a more accurate average duration for phone use. Anthropometric measurements such as cervical spine length, thoracic spine length, lumbar spine length, leg length, foot length, step length, and step width were made. Explanations for these measurements are as follows.

- Cervical spine length: The length between the uppermost and lowermost cervical vertebrae, C1–C7, was measured in the standing position.
- Thoracic spine length: The length between the uppermost and lowermost thoracic vertebrae, T1–T12, was measured in the standing position.
- Lumbar spine length: The length between the uppermost and lowermost lumbar vertebrae, L1–L5, was measured in the standing position.
- Leg length: The distance between the anterior superior iliac spine and the medial malleolus was measured while standing.
- Foot length: In an upright position, with equal weight on the feet, the foot length was measured by drawing from the tip of the longest toe in front and the heel in the back.
- Step width: Participants were stopped while walking, and the distance between their steps was measured.

All anthropometric measurements were performed using a standard anthropometric toolkit, including a stadiometer for height, a non-stretchable measuring tape for lengths, and anthropometric calipers for segmental measurements. Participants were included in the study if they met the following criteria: aged 18–25, had no history of neurological or musculoskeletal disorders, had not experienced fall-related injuries in the past two years, and voluntarily agreed to participate. Exclusion criteria included a diagnosed postural disorder, a history of spinal surgery or trauma-related physical limitations, or any condition that would interfere with proper sensor placement (e.g., skin issues, open wounds, or implanted devices in the spine region).

### 2.3. Statistical Analysis

The SPSS for Windows Version 27 statistics program was used for data analysis. The variables obtained from the measurements were presented as mean ± standard deviation. Normality was assessed using the Shapiro–Wilk test and homogeneity of variances using Levene’s test. When variances were unequal, Welch’s *t*-test was used. In comparisons between groups separated as male and female, the *t*-test for the group was applied in cases where parametric conditions were met, and the Mann–Whitney U test was used in cases where they were not met. Sex-based comparisons were pre-specified because anatomical and behavioral differences can influence spinal curvature and wearable-sensor ergonomics. Significance was evaluated with two-sided α = 0.05. Effect sizes were calculated as Cohen’s d for parametric tests and rank-biserial correlation for nonparametric tests. To aid interpretation with a small sample, 95% confidence intervals were reported alongside *p* values. The chi-square test was used to compare categorical data.

### 2.4. Sensors and Data Collection

In this study, 9-axis IMU sensors were preferred for use in the movement and posture analysis of the spine. MPU-9250 9-axis IMU sensors were used in the system design. It was integrated with a low-power Bluetooth BLE 5.0 data acquisition system. This integration transferred the obtained data to the computer environment in real time and recorded it. The sensors consist of three main components: accelerometer, gyroscope, and magnetometer. These components can measure changes in the x, y, and z axes. The accelerometer can detect linear movements with a sensitivity range of ±2 g to ±16 g, while the gyroscope can measure angular velocity with a range of ±250°/s to ±2000°/s. In addition, the magnetometer can track fixed positions relative to the Earth’s magnetic field.

During the raw data processing phase, the acceleration, gyroscope, and magnetic field measurements were made meaningful using the Savitzky–Golay filter. In this way, more accurate and consistent results were obtained. Due to their low cost and portability, IMU sensors offer a more complementary and practical alternative to expensive and invasive traditional imaging methods (such as X-ray and MRI). Instant data transmission via Bluetooth allowed users to correct their incorrect posture quickly.

The sensors were placed on five vertebrae along the spine: C1, C7, T5, T12, and L5. This placement provided the comprehensive monitoring of spinal movements from the cranium and cervical vertebrae to the pelvis. For example, sensors placed in the C1 region recorded detailed kinetics of the top of the head and upper cervical spine; C7 sensors recorded movements in the transition area between the cervical and thoracic spine; T5 sensors recorded rotation and lateral flexion movements of the trunk; T12 sensors recorded movements in the thoracolumbar transition area; and L5 sensors recorded movements in the pelvis and lower body.

**Figure 2:**
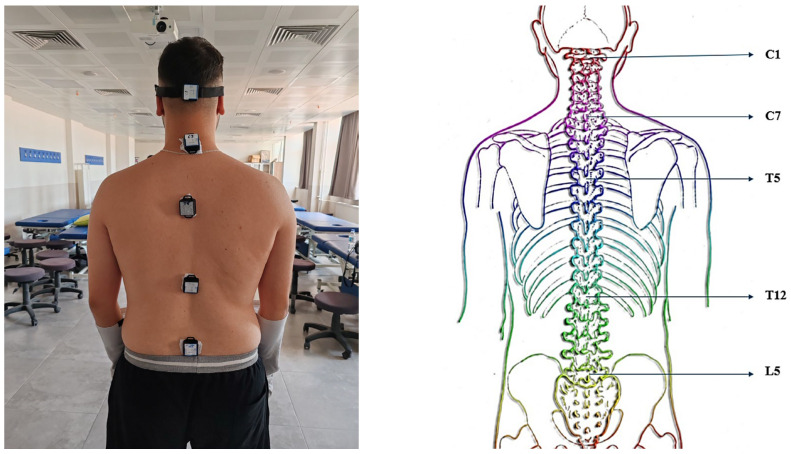
Location of sensors and corresponding spinal segment definitions. IMUs were placed at C1, C7, T5, T12, and L5 vertebral levels. These placements defined the following posture-related segments: C1–C7 (cervical lordosis), C7–T12 (thoracic kyphosis), and T12–L5 (lumbar lordosis) in the sagittal plane, and C1–L5 lateral deviation in the coronal plane (scoliosis).

The sensors were fixed to the body using medical tape and elastic fixators to reduce skin artifacts and alignment errors. Sensors were calibrated by anatomical alignment prior to placement, with fixed bony landmarks ensuring consistent positioning across participants. To minimize misalignment and placement errors, sensors were positioned relative to spinous processes (C1, C7, T5, T12, and L5) by the same trained investigator. Each sensor was manually aligned with the sagittal, coronal, and transverse planes during placement. Elastic straps and medical tape were used to prevent sensor tilt or displacement during movement. Prior to recording, sensors were zeroed in the upright posture to reduce orientation bias. Drift and noise were minimized using Madgwick’s filtering and repeated calibration checks before each trial. Thus, misalignments were prevented, and the sensors were ensured to work correctly. The placement of the sensors at the specified points is shown in Figure 2. As illustrated in Figure 2, the sensor placement defines specific spinal segments that were used consistently across all analyses: C1–C7 for cervical lordosis, C7–T12 for thoracic kyphosis, T12–L5 for lumbar lordosis, and lateral (*Y*-axis) deviation across C1–L5 for scoliosis. These definitions were selected to align with established radiographic standards in spinal biomechanics.

All IMU sensors (MPU-9250) were sampled at 100 Hz. Sensors were synchronized by simultaneous initialization and timestamp alignment. Raw orientation data were processed in local sensor frames and transformed into an anatomical global frame defined by the upright reference posture. A neutral standing trial was used to initialize zero reference angles. The orientation was estimated using the Madgwick filter (β = 0.1), combining accelerometer, gyroscope, and magnetometer signals with prior static gyroscope bias correction and magnetometer calibration. Gravity alignment and heading drift compensation were applied before Euler angle conversion. Relative quaternions between adjacent sensors provided sagittal (flexion/extension), coronal (lateral bending), and axial (rotation) measures. Time series data were smoothed using a Savitzky–Golay filter (window length 11 samples, polynomial order 3). Additional raw-to-processed signal examples are provided in Supplementary Figure S1.

This study calculated angular changes in various parts of the spine using data obtained from IMU sensors placed on the spine. The data obtained from the sensors are processed using Euler angles and quaternions. This data allows the tracking of angular changes over time. Movement patterns in the cervical, thoracic, and lumbar regions were analyzed. Movement data were analyzed throughout the time series to examine differences in spine inclination angles, segmental alignment, and position changes.

### 2.5. Machine Learning (ML) Methods

This study applied ML methods to model IMU-derived proxy deviation labels using demographic, anthropometric, and daily behavior variables. Before modeling, feature vectors were standardized with z-scores within each cross-validation fold to avoid data leakage. Predictors included age, sex, height, weight, BMI, weekly physical activity, and daily desk/smartphone use, while IMU-based posture angles were used to generate outcome labels. Feature selection was primarily based on the literature-driven inclusion of demographic, anthropometric, and behavioral predictors. No automated dimensionality reduction (e.g., PCA, RFE) was applied due to the small sample size; instead, predictors were pre-specified a priori and all retained in the models. This ensured interpretability and avoided overfitting. To address the small sample size *(n* = 30) and severe class imbalance, model evaluation was redesigned to include leave-one-subject-out (LOSO) and stratified k-fold cross-validation. Oversampling techniques (SMOTE or ROS, depending on the minority sample size) and *class_weight* adjustments were applied. For each validation scheme, 95% confidence intervals were calculated using Wilson’s method. The preprocessing pipelines and tuned hyperparameters for each algorithm are summarized in Table 2.

ML is a sub-branch of artificial intelligence that focuses on learning from data and making predictions. This study aimed to obtain the best results using different ML algorithms. The methods were evaluated using performance metrics such as accuracy, precision, recall, and F1 score. In the anatomic posture analysis and estimation process, six different ML algorithms were used in this study to evaluate the effect of various data structures and features on modeling. The performance of each algorithm was analyzed using metrics such as accuracy, precision, recall, and F1 score. The formulas of these metrics are as follows:

- Accuracy: The ratio of the examples the model correctly classified to the total examples.

$$Accuracy=\frac{TP+TN}{TP+TN+FP+FN}$$

- Precision: Indicates how many examples classified as positive are positive.

$$Precision=\frac{TP}{TP+FP}$$

- Recall: Indicates how many true positive examples were correctly predicted.

$$Recall=\frac{TP}{TP+FN}$$

- F1 Score: The harmonic mean of precision and recall.

$$F1=\frac{2\times Precision\times Recall}{Precision+Recall}$$

TP, TN, FP, and FN, used in ML, are terms used to evaluate the performance of a classification model. These metrics express whether the model’s predictions are correct or incorrect. Table 3 provides explanations of these terms.

## 3. Results

The demographic characteristics, daily living activities, and anthropometric measurements of 15 males and 15 females were compared by sex. Women reported longer physical activity (*p* < 0.05) and showed a non-significant trend toward longer standing time (*p* = 0.053), whereas men had a greater cervical spine length and leg length (*p* < 0.05; Table 4). For each comparison, *p* values are presented alongside effect sizes (Cohen’s d for approximately normal variables; rank-biserial correlation otherwise) to aid interpretation in a small sample (*n* = 30). Although several variables (e.g., height, leg length) showed relatively large absolute differences, high within-group variability reduced statistical power and some contrasts did not reach significance. Accordingly, the sex group contrasts in Table 4 should be interpreted descriptively with effect sizes rather than as definitive differences in this small sample. Conversely, physical activity and standing time exhibited consistent between-group trends with lower variability, yielding statistically significant results despite modest mean differences. These findings should be interpreted descriptively and with caution given the sample size.

The study used dynamic posture data obtained from IMU sensors placed on the participants’ C1, C7, T5, T12, and L5 vertebrae. The angular changes in the transition from the upright to the normal posture were analyzed with nine-axis IMU sensors placed at five main points. Raw data were collected using each sensor. The participants were first asked to stand in an upright posture and then to move to postures where they perform daily activities that they are comfortable with. Postural analyses were performed by observing the angular changes between the upright and relaxed positions. Three-axis angular change values obtained from sensors placed on the spine were used. Specifically, *X*-axis changes for cervical lordosis (C1–C7), thoracic kyphosis (C7–T12), and lumbar lordosis (T12–L5), and *Y*-axis changes for scoliosis were calculated.

In this study, posture-related deviation categories were derived from IMU-estimated angles relative to reference ranges from the literature. Cervical lordosis deviation was labeled as increased if the C1–C7 *X*-axis angle > 45° and decreased if <35°. Thoracic kyphosis deviation was labeled as increased if the C7–T12 angle > 40° and decreased if <20°. Lumbar lordosis deviation was labeled as increased if the T12–L5 angle > 60° and decreased if <40°. Scoliosis screening deviation was labeled when lateral *Y*-axis deviation exceeded ± 10°.

Following the preprocessing and calibration described in the Methods Section, triaxial angle changes were computed and summarized. Table 5 evaluates the deviations of cervical lordosis, thoracic kyphosis, lumbar lordosis, and scoliosis according to gender. No statistically significant difference was found between women and men (*p* > 0.05).

As shown in Table 4, the absolute differences were large for some anthropometric variables (such as height or leg length) and statistical significance was not achieved due to high inter-individual variation. Conversely, significant results were obtained for some variables, such as the duration of physical activity, due to the more pronounced inter-group trend, although the differences were more minor. This is due to statistical variables such as sample size and data distribution.

**Figure 3:**
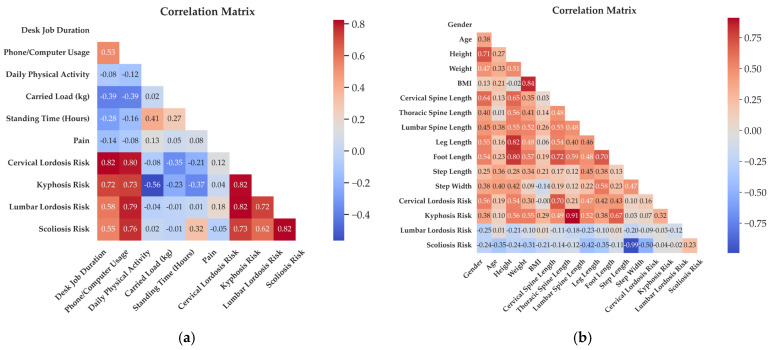
(**a**) Correlation matrix between activities of daily living and cervical lordosis, thoracic kyphosis, lumbar lordosis, scoliosis, and pain. (**b**) Correlation matrix between demographic characteristics, anthropometric measurements, cervical lordosis, thoracic kyphosis, lumbar lordosis, and scoliosis.

The relationships between daily living activities, cervical lordosis, thoracic kyphosis, lumbar lordosis, scoliosis, and pain were examined in the correlation matrix given in Figure 3a. Because multiple pairwise correlations were explored for a small cohort, the results were interpreted with caution. False discovery rate (FDR) correction was applied to control for multiple testing, and only associations remaining significant after adjustment were considered in the discussion. Cervical lordosis, thoracic kyphosis, lumbar lordosis, and scoliosis deviation categories were defined by angle changes that exceed the expected values. The analyses included correlation and regression analyses to evaluate the effects of daily living habits on different posture segments. A negative correlation increases as you move towards the blue color, and a positive correlation increases as you move towards the red color. In Figure 3b, the relationships between demographic characteristics, such as gender, age, height, weight, and BMI, and anthropometric measurements, such as vertebral column length (cervical, thoracic, and lumbar spine length), leg length, foot length, step length, step width, and cervical lordosis, thoracic kyphosis, lumbar lordosis, and scoliosis were examined in the correlation matrix. A negative correlation increases as you move towards the blue color, and a positive correlation increases as you move towards the red color.

### Machine Learning Classification of IMU-Based Deviation Labels

While the cervical lordosis risk, thoracic kyphosis risk, lumbar lordosis risk, and scoliosis risk columns were determined as dependent variables, others were selected as independent variables. During the data preprocessing stage, missing values were identified and filled with the average method, and categorical columns were converted to numerical values. All preprocessing steps (imputation, scaling, and oversampling) were performed strictly within each cross-validation fold: imputation and scaling were fit on the training data only and then applied to the held-out fold, while oversampling (SMOTE/ROS) was conducted exclusively on the training set to avoid data leakage. Then, the data were determined as independent (X) and dependent (y) variables.

The outcome variables in this study were operationalized as risk classifications based on IMU-derived angular estimates, rather than formal clinical diagnoses. Cutoff values were adopted from established reference ranges reported in the spinal bio-mechanics literature (e.g., cervical lordosis 35–45°, thoracic kyphosis 20–40°, and scoliosis risk defined as *Y*-axis deviation >±10°). These ranges have been consistently cited in prior anatomical and postural studies, ensuring compatibility with IMU-based kinematic assessments. Although this study did not include direct validation against radiographic gold standards such as Cobb angle measurements, the selected thresholds reflect values widely recognized in academic sources. Model evaluation was performed using LOSO and stratified k-fold cross-validation instead of a single train–test split. To address severe class imbalance, SMOTE or Random Oversampling (ROS) was applied in combination with class_weight adjustments. For each validation scheme, 95% confidence intervals were calculated.

**Figure 4:**
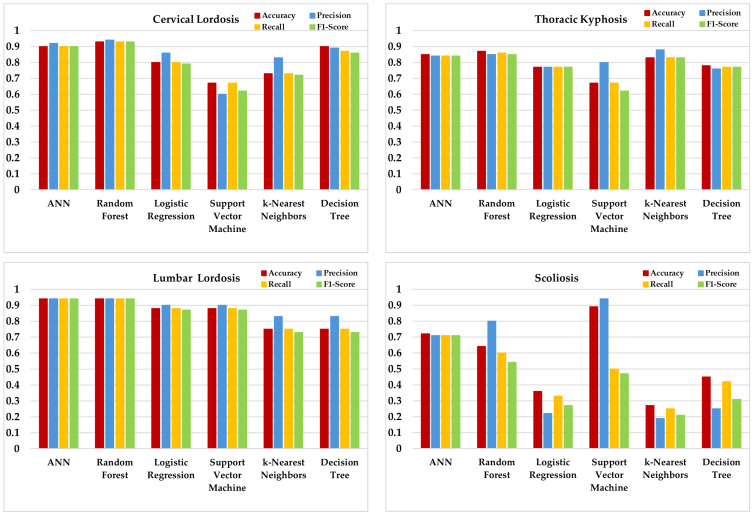
ML algorithm performance results.

**Figure 5:**
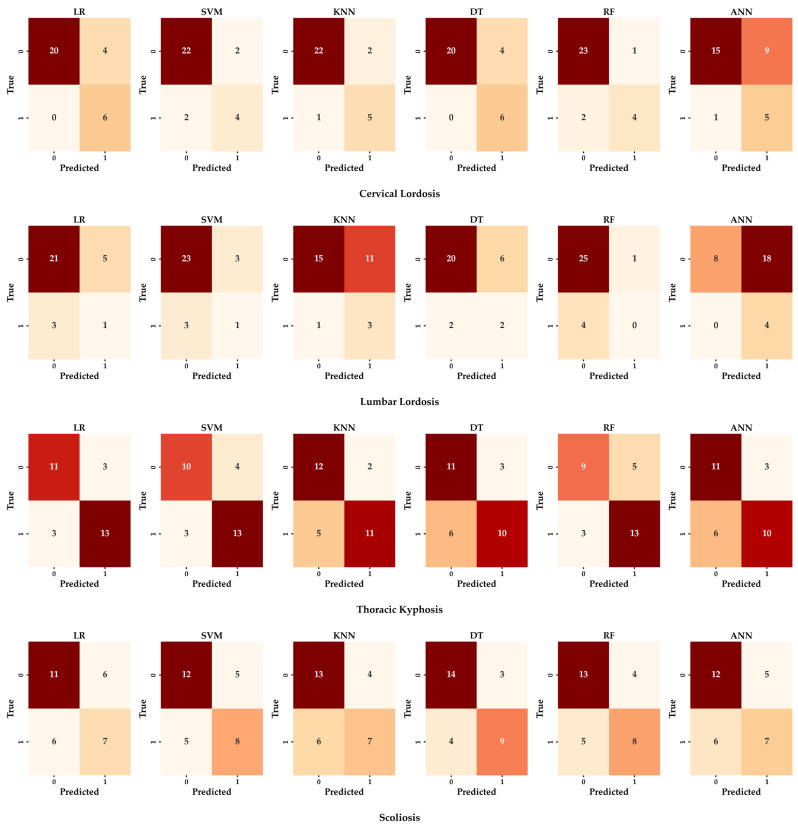
Aggregate confusion matrices of ML algorithms for posture classifications, obtained under stratified 5-fold CV.

Six different ML algorithms were applied and compared for anatomic posture analysis. Logistic Regression (LR), Support Vector Machine (SVM), K-Nearest Neighbor (KNN), Decision Tree (DT), Random Forest (RF), and Artificial Neural Network (ANN) algorithms were evaluated (Figure 4). To address class imbalance and ensure reproducibility, all models were trained within subject-wise LOSO and/or stratified k-fold schemes; per-fold oversampling (SMOTE or ROS) and, where applicable, class_weight = “balanced” were used. Performance was reported with accuracy, precision, recall, F1 score (weighted), and 95% Wilson confidence intervals; fold-level confusion matrices were computed. For scoliosis, balanced accuracy and AUPRC are unstable due to only one positive case; these results are presented descriptively only. Aggregate confusion matrices under the stratified five-fold scheme are shown in Figure 5. Fold-level matrices were not included due to space constraints, but the aggregate results summarize the performance across folds. Scoliosis confusion matrices reflect extremely low positive counts (*n* = 1) and thus provide descriptive rather than confirmatory evidence. Hyperparameters were tuned by stratified k-fold cross-validation on the training folds only, using balanced accuracy as the objective. Per-model settings are given in Table 2.

For cervical lordosis deviation, RF, DT, and ANN achieved a relatively high and balanced performance across metrics, with RF reaching the best balanced accuracy. In thoracic kyphosis, RF and ANN again showed a competitive performance, while KNN provided moderate but consistent results. For lumbar lordosis, RF and ANN remained among the stronger models, although other algorithms showed room for improvement. In scoliosis screening deviation, performance levels were lower and more variable across models, largely due to class imbalance and the complexity of lateral and rotational deviations. In this case, SVM provided the most stable results. However, scoliosis classification should be considered descriptive only, since only one positive case was present in the cohort (Table 5), leading to ≤1 positive in several folds and unstable estimates despite oversampling. Balanced accuracies ranged between 0.55 and 0.82 depending on the model and risk category, while AUPRC values consistently exceeded baseline levels. Majority-class baselines ranged from 0.53 (thoracic kyphosis) to 0.87 (lumbar lordosis). Positive case counts were cervical lordosis = 6, thoracic kyphosis = 16, lumbar lordosis = 4, and scoliosis = 1, with some folds containing ≤1 positive case for scoliosis. These results highlight both the potential and the instability of ML approaches in small and imbalanced samples. Although the accuracy, precision, recall, and F1 score are displayed in Figure 4, interpretation should primarily rely on balanced accuracy and AUPRC due to substantial class imbalance. Majority-class baselines are reported in the Results Section to aid comparison.

## 4. Discussion

This section interprets the main findings, relates them to prior work, and outlines the study limitations and potential clinical applications. The effects of obesity and BMI on kyphosis, lordosis, and scoliosis are remarkable. While height, weight, and BMI are frequently examined in spinal health studies, their direct effects on spinal deformities appear limited. Although there is generally a weak correlation between height, weight, BMI, and spinal deformities such as kyphosis, lordosis, and scoliosis, the significance of this relationship varies between studies. In a study by Rabieezadeh et al. [31], no statistically significant relationship was found between anthropometric measurements and lordosis and kyphosis curvatures in male adolescents aged 12–15. For this reason, they said that height, weight, and BMI are not suitable criteria for estimating cervical lordosis and kyphosis angles in men in this age group. Lazic et al. [32] stated that cervical lordosis changes with age and differs between genders. They stated that women have higher cervical lordosis and vertebral body height, while men have more expansive intervertebral spaces. Tao et al. [33] emphasize that cervical lordosis is affected by intervertebral disks and various vertebral anatomical factors rather than anthropometric measurements. Yiwa et al. [34] stated that a higher BMI contributes to increased kyphosis because it will cause additional pressure on the spine. Still, this relationship was not statistically significant in their study. Bayartai et al. [35] stated that children and adolescents with obesity have significantly larger thoracic kyphosis angles than those with a healthy weight and that obesity adversely affects spinal posture and mobility. Valdovino et al. [36] showed that excessive weight can negatively affect sagittal alignment and vertebral growth. Parmitha et al. [37] revealed a relationship between BMI and vertebral rotation deformity in adolescents with idiopathic scoliosis. Warren et al. [38] stated that even a healthy BMI affects the risk of developing scoliosis and that factors such as gender and diet contribute to this correlation. Naufal and Azizi [39] reported that BMI significantly affects scoliosis in children aged 4–6. This study found no significant relationship between height, weight, and BMI and cervical lordosis risk, thoracic kyphosis risk, lumbar lordosis risk, and scoliosis risk. It must be emphasized that these findings are exploratory in nature. The cohort comprised only healthy young adults aged 18–25, and the results cannot be generalized to other age groups or to clinical populations. Accordingly, we frame these outcomes as feasibility-level signals rather than definitive diagnostic evidence. It was thought that there was no relationship because the participants had completed their growth period. We believe that different results would have emerged if younger age groups, where bone development was not complete, were included in this study. This is one of the shortcomings of this study. Although the current study was conducted on healthy individuals, the term “risk” refers not to a current diagnosis but to deviations from clinically accepted reference angles. These deviations—though subclinical—may indicate a predisposition to future spinal deformities, especially when combined with poor postural habits or prolonged mechanical stress. The primary aim was to identify early-stage morphological tendencies that might not yet manifest as clinical deformities but represent anatomical misalignments. Therefore, the risk labeling in this study serves as a preventive classification tool rather than a diagnostic conclusion. Future studies including individuals with diagnosed spinal pathologies will help further validate the proposed risk thresholds.

The cervical, thoracic, and lumbar segments affect each other biomechanically. Changes in cervical lordosis are related to thoracic kyphosis and indirectly related to lumbar lordosis. This situation shows compensatory mechanisms between spinal segments [40,41]. In addition, the length, angle, height, and depth of the lumbar lordosis are essential in reducing stress on the thoracic structures, while optimal spinal alignment is achieved by considering the relationships between surgical interventions and vertebral curvatures [42,43,44,45,46]. These findings suggest that the biomechanical balance between spinal segments is of fundamental importance in preventing pain and functional disorders that affect quality of life. Our study measured the lengths of the cervical, thoracic, and lumbar regions. It was determined that as the length of the cervical region increased, the risks of cervical lordosis and thoracic kyphosis increased; as the length of the thoracic region increased, the risk of thoracic kyphosis increased at a very high rate; and as the lumbar length increased, the risks of cervical lordosis and thoracic kyphosis increased.

The biomechanical relationship between different spine regions is essential in understanding and treating spinal deformities. The length, angle, and depth of lumbar lordosis are critical factors affecting thoracic kyphosis and overall spinal alignment [42,43]. Changes in lumbar lordosis, especially in conditions such as obesity, determine the static and dynamic stability of the spine and reduce stress in the thoracic spine [44,45]. In another study, a negative relationship was found between cervical lordosis and thoracic kyphosis, and it was shown that the decrease in cervical lordosis increased thoracic kyphosis [40]. In addition, correcting thoracic kyphosis with surgical interventions also corrects lumbar lordosis, which emphasizes the interconnectedness of spinal segments [41,46]. In this study, in line with the literature, cervical lordosis, thoracic kyphosis, lumbar lordosis, and scoliosis risks were found to be correlated with others. The findings show that changes in one region of the spine can have cascading effects on other regions and that ensuring proper alignment is vital for optimal spinal health.

Recent studies show that prolonged sitting and excessive smartphone use lead to postural disorders, especially among office workers and young people. Singhvi and Bharnuke’s [47] study showed that prolonged sitting in individuals completing desk work shortens the iliopsoas muscle, which leads to lumbar hyperlordosis. It has been stated that these muscle shortenings increase lower back pain due to increased lordosis. Barut et al. [48] have shown that increased sitting time in desk jobs causes back pain and that these people have higher thoracic kyphosis angles. Studies showing that information technology workers exhibit higher kyphosis and lordotic values than agricultural workers also support these findings [6]. Kim et al. [49] suggested that sitting in office chairs for extended periods may negatively affect lumbar lordotic curvature. In the study of Shivangi et al. [50], it was stated that excessive smartphone use causes a forward head posture, which has a significant relationship with neck disability. Yang et al. [51] stated that inappropriate table heights and poor sitting habits increase the risk of idiopathic scoliosis, especially in adolescents. Still, they reported that changing sitting positions at certain times and ensuring correct posture reduces the likelihood of developing scoliosis. In the studies of Betsch et al. [52] and Brühl et al. [53], it was stated that phone and computer use caused kyphosis and lordosis due to the continuous use of the same position.

In this study, in parallel with other studies, it was found that as the duration of desk work and phone use increased, the risk of cervical lordosis, thoracic kyphosis risk, lumbar lordosis risk, and scoliosis risks increased. In addition, it was found that daily physical activity reduced the risk of cervical lordosis and lumbar lordosis, especially thoracic kyphosis.

IMU sensors are used to monitor spinal movements. The 26-sensor system that Valchinov et al. [54] stated in their study is essential in monitoring individual vertebrae and detecting conditions such as kyphosis, lordosis, and scoliosis through Cobb angles. This is of great importance in terms of early diagnosis and treatment planning. Voinea and Mogan [55] evaluate lateral spinal movements using IMU sensors, providing non-invasive scoliosis monitoring. Such innovative approaches are essential in protecting spinal health, especially in young patients.

ML techniques can predict spinal curvatures with very low error. Mak et al.’s [56] study shows that neural networks can detect spinal deformities with an error of 0.261 cm. Similarly, Cho et al.’s [57] study showed that SVM techniques can detect scoliosis-related gait changes with 90.5% accuracy. Balaji et al. [58] demonstrated a mean sensitivity (mAP) of 98.5% with 96.8% sensitivity and 97.2% recall in ML used to detect and grade lumbar spondylolisthesis using X-ray images automatically. This indicated that ML is a reliable and effective solution for the early detection of lumbar spondylolisthesis.

Spinal posture was analyzed using IMU sensors, and classification was performed with ML algorithms. ML analyses indicated that RF and ANN consistently achieved a strong and balanced performance across cervical and lumbar lordosis risk prediction, while DT also performed competitively in cervical outcomes. For thoracic kyphosis, RF and ANN again showed a robust performance, whereas KNN provided moderate but stable results. In contrast, scoliosis risk prediction yielded more variable outcomes; SVM achieved the most reliable performance, but precision and recall values fluctuated due to class imbalance and the rotational nature of scoliosis. These findings suggest that sagittal plane deviations (lordosis, kyphosis) can be captured more reliably by wearable IMU sensors, whereas coronal and rotational deviations (scoliosis) remain technically challenging. The overall classification accuracy across outcomes ranged between 0.60 and 0.90, depending on the model and risk category. This underscores both the potential and the limitations of ML approaches in small, imbalanced datasets. Importantly, the “risk” labels in this study represent subclinical deviations from normative posture rather than clinical diagnoses, suggesting their potential utility as preventive indicators rather than diagnostic endpoints. However, significant fluctuations were observed in model performances in scoliosis prediction. These inconsistencies were especially apparent in coronal plane predictions such as scoliosis. Unlike sagittal movements, which are typically larger in amplitude and more easily captured by IMU sensors, coronal and rotational deviations tend to be subtle and multidimensional. This increases their sensitivity to minor sensor misalignments, soft tissue movement, and measurement noise—particularly along the *Y*-axis. Therefore, the performance variation observed across models in coronal parameters is likely due, at least in part, to the complexity and technical limitations in capturing lateral and rotational spinal movements with wearable sensors. SVM gave the best result for scoliosis prediction. On the other hand, there were inconsistencies in precision and recall values. This indicates that additional datasets or alternative modeling approaches should be evaluated for scoliosis prediction. Our study provides a feasibility analysis where IMU-derived posture angles were used to define proxy risk labels and ML classifiers explored their associations with demographic, anthropometric, and daily habit variables. These findings are exploratory and should not be interpreted as a clinical diagnosis. In this context, we suggest that our study contributes to the literature.

### 4.1. Limitations

This study has several limitations. Firstly, the risk categories represent IMU-derived proxy labels based on thresholds from the literature rather than clinical diagnoses such as radiographic Cobb angles. The thresholds were adopted from reference ranges commonly reported in spinal biomechanics studies and are compatible with IMU-based angular estimates, but were not directly validated against gold-standard clinical measures (e.g., radiographs, optical systems). As such, the present findings should be regarded as preliminary indicators of postural deviations, not as diagnostic outcomes. Additionally, although participants were encouraged to verify their daily phone and computer usage through screentime applications, not all individuals were able to provide objective logs. As a result, part of the dataset still relied on self-reported estimates, which may introduce recall bias. Future studies should consider the mandatory integration of smartphone usage tracking or wearable monitoring devices to obtain fully objective measures of daily activities.

Secondly, although demographic and anthropometric features were included as auxiliary predictors in the ML stage, the foundation of the classification task lies in the IMU-derived outcome categories, in line with other IMU-based approaches [20,27]. This hybrid design reflects clinical practice, where sensor-based biomechanical assessments are interpreted alongside patient characteristics. Sensor-to-anatomy alignment error was not quantified using inter-rater or test–retest ICCs in this study. All placements were performed by the same investigator using palpable landmarks and neutral standing calibration to minimize variability. Future studies should include formal reliability testing.

Finally, the small sample size (*n* = 30) and severe class imbalance—particularly for scoliosis risk—limit the generalizability of the results. The reported model performances should therefore be interpreted as exploratory feasibility outcomes, and validation in larger, balanced cohorts with direct clinical references will be necessary to confirm the accuracy and clinical utility of the proposed IMU-based risk definitions. In particular, the scoliosis classification was underpowered (1/30 positives), resulting in a fold-wise instability that cannot be corrected by oversampling; therefore, the scoliosis results should be regarded as exploratory in nature and should be discussed qualitatively.

### 4.2. Clinical Applications

In practice, IMU-based posture monitoring may serve as a supportive adjunct to standard assessments by potentially enabling longitudinal, out-of-clinic tracking in workplaces, schools, and home settings. Possible future applications could involve exploratory use in ergonomic screening, the monitoring of daily postural habits, or providing feedback to encourage behavior change. Such uses remain hypothetical and would require prospective validation against clinical standards and the establishment of clear clinical thresholds before any diagnostic implementation can be considered. Accordingly, the current workflow should be regarded as an exploratory and non-diagnostic tool that may inform future preventive strategies.

## 5. Conclusions

The results support the feasibility of deriving IMU-based angle-defined proxy risk labels and exploring their associations with demographic, anthropometric, and daily habit variables; however, this work does not establish clinical effectiveness. Effectiveness can only be demonstrated through head-to-head validation against traditional clinical standards (e.g., radiographic Cobb angles or validated optical systems) in larger and more diverse cohorts.

Future studies should include direct clinical validation, balanced and larger samples with subject-wise cross-validation and confidence intervals, and an evaluation of models that also leverage IMU time series signals. Until such evidence is available, the proposed workflow should be considered a complementary research tool rather than a stand-alone diagnostic.

## Figures and Tables

**Table 1:** Power analysis targets: no skill accuracy (p_0_) and required accuracy (p_1_) for 80% power per outcome.

Target	No Skill Accuracy (p0)	Required Accuracy (p1) for 80% Power
Cervical Lordosis	0.80	0.96
Thoracic Kyphosis	0.53	0.77
Lumbar Lordosis	0.87	0.99
Scoliosis	0.57	0.80

**Table 2:** Preprocessing pipelines and tuned hyperparameters for each ML algorithm.

Algorithm	Preprocessing	Hyperparameters
SVM	StandardScaler → (SMOTE if minority ≥3, else ROS)	kernel = {rbf, linear}; C = {0.1, 1, 10, 100}; γ = {scale, 0.1, 0.01, 0.001}; class_weight = balanced
Logistic Regression	StandardScaler → (SMOTE/ROS)	solver = liblinear; penalty = {l1, l2}; C = {0.01, 0.1, 1, 10, 100}; class_weight = balanced; max_iter = 2000
KNN	StandardScaler → (SMOTE/ROS)	n_neighbors = {1, 3, 5, 7, 9}; weights = {uniform, distance}; metric = minkowski; *p* = {1, 2}
Decision Tree	(SMOTE/ROS)	criterion = {gini,entropy}; max_depth = {None, 2–5}; min_samples_split = {2–5}; min_samples_leaf = {1–3}; max_features = {None, sqrt, log2}; ccp_alpha = {0.0, 0.001, 0.01}; class_weight = balanced
Random Forest	(SMOTE/ROS)	n_estimators = 100; max_depth = None; random_state = 42; class_weight = balanced
ANN	StandardScaler → (SMOTE/ROS)	hidden_layer_sizes = {(8), (16), (32), (16,8), (32,16)}; activation = {relu,tanh}; alpha = {0.0001, 0.001, 0.01}; learning_rate = adaptive; learning_rate_init = {0.001, 0.01}; solver = adam; max_iter = 1000; early_stopping = True; n_iter_no_change = 20; random_state = 42

**Table 3:** Explanations of terms.

Abbreviation	Description	Meaning
TP	True Positive	Situations that the model predicts are positive and are positive.
TN	True Negative	Situations that the model predicts are negative and are negative.
FP	False Positive	Situations that the model predicts are positive but are negative (type I error).
FN	False Negative	Situations that the model predicts as negative but are positive (type II error).

**Table 4:** Examination of demographic characteristics, daily habits, and anthropometric measurements by gender.

Demographic Characteristics Daily Living Activities Anthropometric Measurements	Male (Mean ± SD)	Female (Mean ± SD)	Test	*p* Value	Effect Size
Age (years)	21.00 ± 1.46	20.40 ± 1.45	*t*-test	0.27	0.41
Height (cm)	175.40 ± 5.95	160.40 ± 4.34	*t*-test	0.001 *	2.88
Weight (kg)	70.93 ± 13.64	59.67 ± 12.57	*t*-test	0.026 *	0.86
BMI	22.98 ± 3.79	23.24 ± 5.16	Mann–Whitney U	0.803	−0.06
Desk Time (h/day)	2.67 ± 1.88	1.80 ± 1.07	Mann–Whitney U	0.309	0.57
Phone/Computer Time (h/day)	5.00 ± 2.88	3.63 ± 1.45	Mann–Whitney U	0.26	0.6
Physical Activity (h/day)	1.59 ± 0.69	3.53 ± 2.22	Mann–Whitney U	0.014 *	−1.18
Standing Time (h/day)	3.53 ± 1.64	5.10 ± 2.49	*t*-test	0.053	−0.74
Cervical Spine Length (cm)	9.97 ± 1.32	7.90 ± 1.04	*t*-test	0.001 *	1.74
Thoracic Spine Length (cm)	28.93 ± 4.50	25.30 ± 2.68	*t*-test	0.013 *	0.98
Lumbar Spine Length (cm)	13.13 ± 2.70	11.13 ± 1.52	*t*-test	0.02 *	0.91
Leg Length (cm)	91.47 ± 4.85	84.73 ± 3.65	Mann–Whitney U	0.001 *	1.57
Foot Length (cm)	28.07 ± 2.02	25.27 ± 1.44	*t*-test	0.001 *	1.6
Step Length (cm)	48.40 ± 13.91	42.67 ± 18.38	*t*-test	0.344	0.35
Step Width (cm)	6.47 ± 4.00	3.87 ± 2.19	*t*-test	0.038 *	0.81

* *p* < 0.05 was considered statistically significant; *p* ≥ 0.05 was considered not significant (ns).

**Table 5:** Cervical lordosis, thoracic kyphosis, lumbar lordosis, and scoliosis risks according to gender.

	Male (Mean ± SD)	Female (Mean ± SD)	*p* Value
Cervical Lordosis Yes No	5 10	1 14	0.068
Thoracic Kyphosis Yes No	7 8	9 5	0.064
Lumbar Lordosis Yes No	1 14	3 12	0.28
Scoliosis Yes No	0 15	1 14	0.30

## Data Availability

The data associated with this study are not publicly available due to ethical restrictions. However, they may be provided by the authors upon reasonable request and in accordance with the conditions approved by the institutional ethics committee. In addition, the analysis code is openly available at https://github.com/ybahadirkoca/Posture_Analysis_ML (accessed on 16 September 2025). Raw data may be provided by the authors upon reasonable request and in accordance with the conditions approved by the institutional ethics committee.

## Supplementary Materials

The following supporting information can be downloaded at: https://www.mdpi.com/article/10.3390/s25195963/s1, Figure S1: Representative raw-to-processed IMU time series segments and preprocessing steps.
