# Supplementary figures and images for: Anatomy-Based Assessment of Spinal Posture Using IMU Sensors and Machine Learning
Source: Sensors (Basel). 2025 Sep 25;25(19):5963. doi: 10.3390/s25195963 (PMC12526714; doi:10.3390/s25195963)

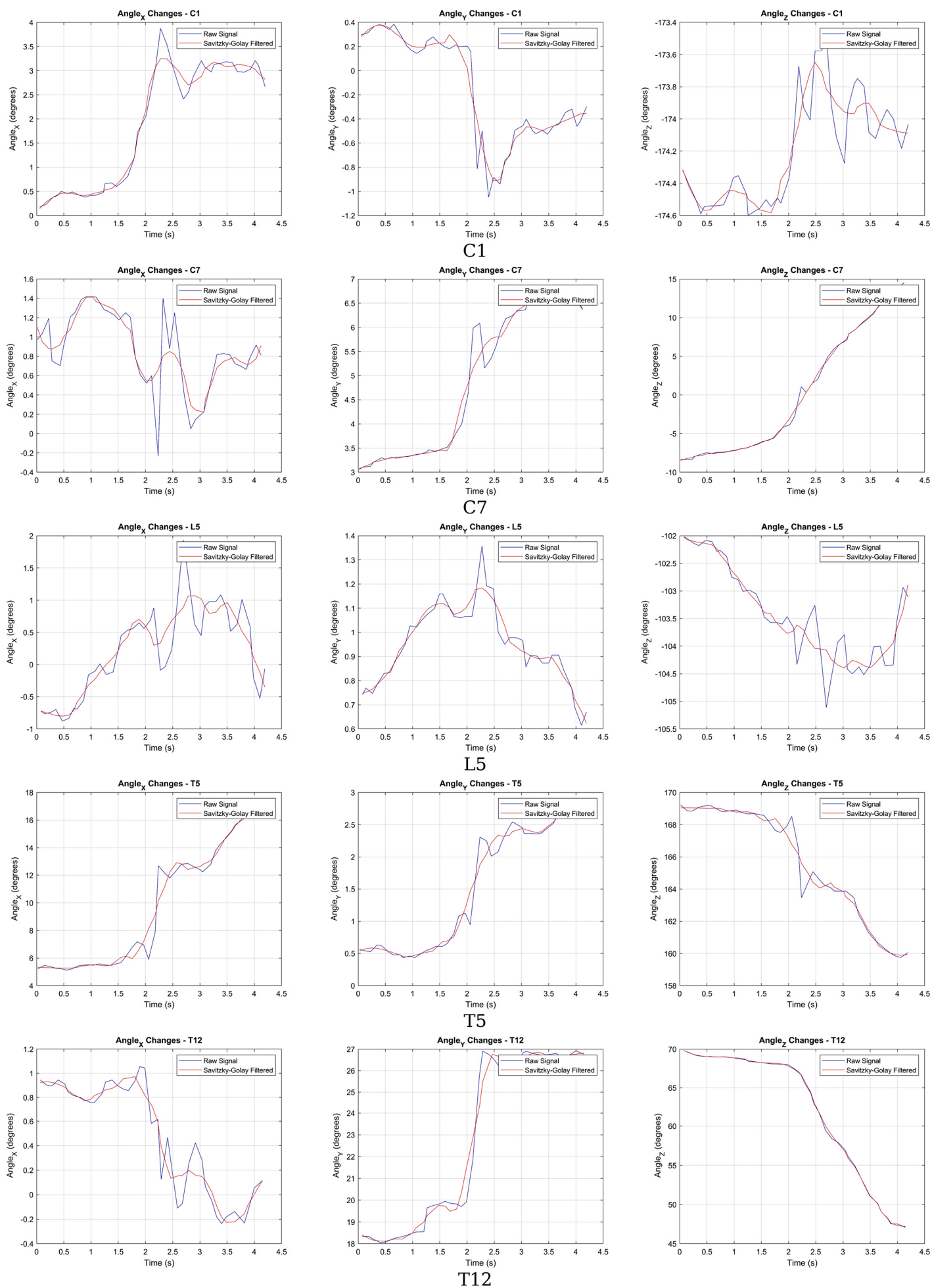

**Figure S1** Representative raw-to-processed IMU time-series segments and preprocessing steps.

Supplement: Supplementary file 1 [file sensors-25-05963-s001.zip › sensors-3817979-supplementary.pdf]
